# 

**Published:** 1890-11-08

**Authors:** 


					KIlVIll CHEMISTS, GROCERS^ ScT,
W W IIIL SKSSffiSBSKiS ess^ WW ^ Sf^gSI"^ throughout
* " ?? ? ?? the disease unler PW^ U ?hich thej w?re THE UNITED KINGDOM.
hesitation to advise you
your clients who have mental exertions, to use Johnston'
tonic and a palatable food."?J. M. BEAUSOLIEL, M.D.
No. 215. Vol IX.] Bat-today,
November 8th, 1890.
[One Penny.

				

